# School Adjustment and Socio-Family Risk as Predictors of Adolescents’ Peer Preference

**DOI:** 10.3389/fpsyg.2021.645712

**Published:** 2021-07-21

**Authors:** Yolanda Sánchez-Sandoval, Laura Verdugo

**Affiliations:** Departamento de Psicología, Instituto de Investigación e Innovación Biomédica de Cádiz, Universidad de Cádiz, Cádiz, Spain

**Keywords:** adolescents, social preference, sociometric status, school adjustment, socio-family risk

## Abstract

This work analyzes peer preferences at the beginning of adolescence. For this purpose, each adolescent’s sociometric status was studied in their classroom group, and attempts were made to identify indicators of academic, personal, and socio-family adjustment related to that status. Participants were 831 adolescents studying 1st grade of Compulsory Secondary Education (CSE), in 31 classrooms from 10 schools. The 31 tutors of these students also participated. Sample selection was intentional. A quantitative research approach was used. Sociometric data were collected using the nomination method. Teachers provided information about these youths’ adjustment and family risk variables. Descriptive analyses and bivariate correlations were calculated as a preliminary analysis of the study. Chi-square tests or ANOVAs examined the similarities and differences between status based on personal, socio-family, and school adjustment variables. Lastly, linear regression analysis and a Structural Equation Model (SEM) were performed. These latter analyses revealed that good performance and academic adjustment are important predictors of successful social relations. Also, the data show that the presence of personal and socio-family risk variables makes it difficult for adolescents to be accepted by their peers. The results suggest the need for school and family support to promote peer acceptance. Working on both aspects can help improve classroom coexistence. Intervention techniques are recommended for the entire group to intervene on attitudes, interpretations, and behaviors that enrich individual tools and the collective climate.

## Introduction

Peer relationships in the school context represent a unique experience of socialization during adolescence. These relationships are more egalitarian and transient than family relationships and include a wide range of phenomena, such as behaviors, affects, thoughts, motivation, and relationships (Rubin et al., 2015). Maintaining adequate relationships with peers has multiple benefits for individuals’ cognitive, affective, and social development (Becker and Luthar, 2002; Buhs et al., 2006).

Many researchers have analyzed the concept of peer preference (or social preference among peers) and have tried to determine its correlates during adolescence. Peer preference in the classroom has been widely assessed using sociometric techniques. Sociometric nomination expresses the position or social status that the student presents in their classroom, according to their peers’ opinion (Aparisi et al., 2015). The nomination method is one of the most common procedures for measuring sociometric status (García-Bacete, 2006; Muñoz et al., 2008). Each student names the classmates they like to be with and those they do not like to be with. It is thus possible to prepare for each student a social preference index (the number of positive choices minus the number of negative choices) and a social impact index (the amount of positive and negative choices) (Coie et al., 1982; Coie and Dodge, 1983). These indices allow classification of the students into different sociometric statuses: popular (positive social preference and high impact), rejected (negative social preference and high impact), neglected (low social preference), controversial (average social preference and high social impact), or, finally, the rest of adolescents as average. Being popular is even a primary goal of many adolescents to gain greater social security (Igbo and Nwaka, 2013). In contrast, experiences of social rejection can affect adolescent well-being, even promoting the onset of psychiatric disorders (Schneider et al., 2016). The quality of relationships and the degree of acceptance/rejection experienced by adolescents are key aspects of psychosocial adjustment at this life stage (Estévez et al., 2009).

According to Bronfenbrenner (1979) ecological model of human development, what happens in the peer microsystem is not alien to the experiences in other systems (such as family and school) that are significant for the developing individual. This study focuses on the links between adolescents’ acceptance by peers and variables from the school microsystem and the family microsystem.

Researchers have tried to identify the characteristics of boys and girls of each sociometric status. There is considerable empirical evidence regarding the involvement of variables from the school microsystem. School adjustment, understood as the adaptation to the demands and characteristics of the school system as well as the degree to which adolescents feel comfortable in this setting, and which we analyze in this work, is one of the variables that differentiate the students (Rodríguez-Fernández et al., 2012). Recent studies have confirmed the association between peer acceptance and academic performance (Gallardo and Barrasa, 2016; Rambaran et al., 2016; Wentzel et al., 2020). Academic difficulties and school failure are generally higher among rejected students. Students nominated negatively by their peers are more likely to attribute academic failure to internal causes such as ability and effort than positively nominated students (Inglés et al., 2017). This greater school failure is due to different factors (Cava and Musitu, 2000). On the one hand, social acceptance influences motivation for academic achievement and participation in learning tasks. Second, some personal skills are involved both in socially competent behavior and academic adjustment. These skills include, for example, self-regulation, impulse control, helping and cooperative behaviors, or self-confidence. And finally, as the evaluations made by teachers and peers usually coincide (Ahn and Rodkin, 2014), the same children who are evaluated more negatively are habitually the ones who receive less support, both by teachers and peers.

There is less evidence about the role of socio-family variables in peer preferences during adolescence. As indicated in a review on sociometric status and adolescence (Martínez et al., 2012), parents’ support enhances the social competence of their children by increasing their ability to form positive social relationships. This support influences peer acceptance or rejection, with rejected adolescents feeling less supported by their parents, while the accepted children positively value the support and communication with their parents (Estévez et al., 2006). Also, the quality of family relationships is closely related to the behavior that children will develop in social interaction with others in the future (Helsen et al., 2000; Musitu and Cava, 2001; Musitu and García, 2004). Rejected children perceive their families as less cohesive, more conflictive, with less positive communication and achievement orientation, and planning and participation in cultural activities (García-Bacete et al., 1990). Other current researches have shown a different analysis. Studies such as those of Estévez et al. (2014) highlighted the results obtained in their work with adolescents, where they indicated that the emotional and family adjustment levels of aggressive popular students were as negative as those of aggressive rejected students.

The links between social risk and peer preferences are not clear. Van de Schoot et al. (2010) showed that, when sociometric status is analyzed among adolescents at psychosocial risk, there are some differences, and some boys with antisocial behaviors are among the most preferred in the classroom. In our particular study, we will analyze the influence of the accumulation of socio-family risk variables on peer preference among adolescents. According to the cumulative risk theory, when risks accumulate, their effects on well-being increase. The concept of cumulative risk explains that exposure to multiple risk factors predicts more adverse developmental consequences compared to singular risk factor exposure (Evans et al., 2013). As Rodríguez Rodríguez and Guzmán Rosquete (2019) confirmed, the accumulation of at least two risk factors, such as parents’ emotional maladjustment, economic problems in the family, low educational level, and high conflict in family relationships, increase the likelihood of school failure. In the same line, authors such as Garcés-Delgado et al. (2020) have referred to adolescents born and growing up in low-education and low-economic families, with few social support networks, belonging to socially excluded minority cultures and unstructured families as “minors at risk of social exclusion.” The accumulation of potential family risk factors may compromise youth adjustment (Buehler and Gerard, 2013), but the relationship between this variable and peer preference has received less attention.

It is also interesting to know the role of other personal variables. In terms of gender, for example, the percentage of boys rejected by their peers is higher than that of girls (Van de Schoot et al., 2010). The differences between boys and girls remain constant throughout schooling (Martín, 2016). In other research with adolescents, girls perceived higher peer acceptance than boys (Tamm et al., 2014). In line with these data, a recent study on the influence of gender in peer acceptance or rejection at recess has shown that girls and boys both mainly reject boys, in first and second place. The reasons are related to the personality of the rejected classmate, affective characteristics, and differences in the type of game (Luis Rico et al., 2020). Results from other studies have emphasized boys’ poorer development of social skills (Mikas and Szirovitza, 2017). As sociometric status is usually analyzed in peer groups with very similar ages, the age variable has hardly been studied in relation to possible interstatus differences. Despite the little evidence about this variable, we believe it is necessary to analyze possible differences between statuses. When some children are older than their peers in the same grade, it is usually because they have repeated one or more courses. One might wonder whether being older can be considered a risk factor concerning peer preference in the class group. When applying the cumulative risk theory, the presence of multiple personal risk factors (gender, age), together with socio-family risk factors, could lead to more adverse results.

In the present study, we sought to extend our current understanding of the link between peer preference during adolescence and academic functioning, and personal and family variables in students of the first grade of Compulsory Secondary Education (CSE) in Spain. The relationships of academic adjustment with social status among peers have been preferably studied during childhood and, to a lesser extent, during adolescence (Prinstein, 2007). Similarly, little is known about the concrete contributions of family and social contexts of origin to acceptance in the peer group. This work aims to provide new data to research, also replicating the results obtained in other studies in other contexts. Based on the previous findings reviewed, we hypothesized that (1) individual academic adjustment will be positively related to higher peer preference and 2) some personal (being a boy) and family variables (less family involvement and socio-family risk) will hinder adolescents’ preference by their peers.

## Materials and Methods

### Participants

The sample consisted of 831 adolescents enrolled in the 1st grade of CSE in Spain. Data were collected from 31 classrooms of 10 different schools. The 31 tutors of these students also participated. The selection of the sample was done through intentional sampling, taking into account the following parameters: (a) ownership of the school (public schools/private-concerted schools), (b) size of the population (large, more than 90,000 inhabitants/medium, less than 90,000 inhabitants), and c) socioeconomic level of the families (medium/medium-low). To respect the percentages of students in public and private schools in the province of residence, we maintained similar proportions when selecting the schools participating in the sample (75.9% public schools and 24.1% private schools).

All the classes of 1st grade of CSE of the selected schools participated. All the participants who did not have any missing value on the completed scales were selected. Of these participants, 53.5% were boys and 46.5% were girls, the mean age was 12.45 years (SD = 0.706). According to the information of the tutors, 4.7% of students belonged to ethnic minority groups, 11.3% had learning difficulties, 7.2% belonged to unstructured families, 5.3% were described by teachers as having significant economic needs, and at least 1.6% had a member with some substance addiction.

### Measurements

#### Sociometric Status

Following the procedure of Coie and Dodge (1983), sociometric status was calculated following the next steps. To assess *peer liking* (positive nominations), we asked adolescents to nominate three classmates with whom they would like to share experiences and personal needs (e.g., “If you’re worried or have a problem, which classmates would you tell it to and ask for advice?”), to play or spend free time (e.g., “Which classmate do you like to be with the most in your free time [to go out with, at recess,…]?”) and to study with or do homework together (e.g., “Which classmate would you choose to do homework together?”). To assess *peer disliking* (negative nominations), we asked them to nominate three classmates with whom they would not like to do the above-mentioned activities. These nominations were standardized within each classroom. Four scores were calculated for each participant: (1) *standardized* score of the *sum of positive nominations* received, (2) *standardized* score of the *sum of negative nominations* received, (3) *Social Preference Index* (SP), by subtracting the negative nominations from the positive nominations received, and (4) *Social Impact Index* (SI), by adding the positive and negative nominations. Based on these scores, each participant was assigned to one of five sociometric status categories: popular, average, rejected, controversial, and neglected. For this purpose, the following formulas were applied: Popular (Z SP > 1, Z positive nominations > 0, Z negative nominations < 0), Rejected (Z SP < −1, Z positive nominations < 0, Z negative nominations > 0), Neglected (Z SI < −1, Z positive nominations < 0, Z negative nominations < 0), Controversial (Z SI > 1, Z positive nominations > 0, Z negative nominations > 0), and Average (rest of the group). As Cava et al. (2010b) indicate, the internal consistency index (Cronbach’s α) for this measure is rarely used due to theoretical difficulties when conceptualizing sociometric measurement within a classical psychometric framework (Terry, 2000).

#### School Adjustment

Completed by the teacher in the classroom, the Scale of Teacher’s Perception of School Adjustment (EA-P; Cava and Musitu, 1999) is made up of eight items about the teachers’ perceptions of each one of their students in four areas or subscales: Social Adjustment (e.g., “The student’s degree of social adjustment in the classroom”), Academic Performance (e.g., “Current approximate academic performance”), Family Involvement (e.g., “Degree of family implication in the child’s school performance”), and Teacher–Student Relationship (e.g., “His/her relationship with this student”). The response scale ranges from 1 to 10 (1 = *poor/very bad* and 10 = *high/very good*). The reliability of the global scale in this study was α = 0.91, and it was higher than α = 0.85 in the four subscales. These indices are very similar to those used in previous studies, which obtained α = 0.91 or higher (Cava and Musitu, 1999; Cava et al., 2010a).

#### Socio-Family Risk

With the information provided by the teachers on different family variables, the Cumulative Socio-Family Risk Index was generated. The cumulative risks exposure (range 0–4) was calculated by adding the four single risk indicators (0 = *Absence* and 1 = *Presence*): Addictions in the immediate family members, Significant economic needs (serious economic difficulties that impair meeting the basic needs), Very unstructured families or problem families (family group lacking a structure in terms of education, limits, schedules, coexistence, affectivity, and/or families that are in constant conflict), and Ethnicity different from the majority. This kind of score has been used previously to calculate a cumulative socio-family risk index (Rodríguez Rodríguez and Guzmán Rosquete, 2019) and with other psychological constructs, such as adverse childhood experiences (McCrory et al., 2015; Deschênes et al., 2018).

### Procedure

After receiving the consent of the school directors and families, we visited the classrooms. The research project was accepted by the Doctoral Committee of the University of Cádiz (Spain). Permission was obtained from the local educational authorities and the School Council at each school. We obtained informed consent from all individual participants included in the study. Student participation was voluntary. We administered the questionnaires in whole class groups. Tutors were provided with the forms to be completed by them about each of their students.

### Data Analysis

Data analysis was carried out using the Statistical Program for the Social Sciences PASW Statistics for Windows (version 21) and EQS 6.2. We present descriptive results and the relationships between the variables from the bivariate correlation analyses. Based on the standardized Social Preference (SP) and Social Impact (SI) indices, the sociometric status of each participant was calculated. Chi-square tests and ANOVAs were used to analyze the differences and similarities between sociometric status as a function of personal variables (sex and age), socio-family risk variables, and school adjustment variables. The analyses were completed with post hoc group comparisons. Cohen’s *d* was used to calculate the magnitude of the group differences (effect size). The magnitude could be small (*d* = 0.2), medium (*d* = 0.5), or large (*d* = 0.8) (Cohen, 1988).

For the second group of analyses (regression model and Structural Equation Model), the standardized Social Preference score was used as a criterion variable. A regression analysis model was performed to explore the degree to which students’ social preference could be predicted from personal, socio-family variables, and school adjustment variables. Finally, we calculated a Structural Equation Model (SEM) to study the influence of these variables and the degree to which they determine variations in social preference among adolescents.

## Results

Table 1 shows the descriptive statistics of the variables of the study. The results of the Pearson correlation analysis revealed statistically significant correlations between them (*p* < 0.05). Age and the Cumulative Socio-Family Risk Index (CSR) showed a significant negative correlation with the Social Preference Index and the Teacher’s Perception of School Adjustment (and its four subscales: academic performance, social adjustment, teacher–student relationship, and family involvement). Social preference correlated positively with the Teacher’s Perception of School Adjustment.

**TABLE 1:** Descriptive statistics and bivariate correlations between target variables.

Variables	1	2	3	4	5	6	7	8
1. Age	1							
2. CSR	0.21**	1						
3. Social Preference	−0.13**	−0.13**	1					
4. Academic Performance	−0.37**	−0.30**	0.33**	1				
5. Family Involvement	−0.28**	−0.28**	0.20**	0.71**	1			
6. Social Adjustment	−0.22**	−0.26**	0.37**	0.60**	0.48**	1		
7. Teacher–student relationship	−0.16**	−0.20**	0.28**	0.60**	0.59**	0.65**	1	
8. EA-P	−0.32**	−0.32**	0.35**	0.88**	0.85**	0.79**	0.82**	1
*M*	12.45	0.18	0.00	5.64	6.53	6.71	7.13	6.49
*SD*	0.70	0.50	0.98	2.24	2.35	1.77	1.64	1.69

CSR, Cumulative Socio-Family Risk Index; EA-P, Scale of Teacher’s Perception of School Adjustment. **p < 0.01.

### Sociometric Status

Taking into account the positive and negative preferences received by each boy and girl from their classmates, the standardized Social Preference and Social Impact indices of each student were calculated. With these data, each of the 831 participants was classified in one of the five sociometric statuses. Descriptive data of the five groups are shown in Table 2.

**TABLE 2:** Distribution and descriptive statistics of the sociometric status.

	Rejected	Controversial	Neglected	Average	Popular	Total
Frequency	73	32	55	594	77	831
Percentage	8.8	3.9	6.6	71.5	9.3	
**Preference**						
Mean	−2.22	−0.81	−0.03	0.15	1.35	0.00
SD	(1.08)	(1.27)	(0.18)	(0.45)	(0.37)	(0.98)
**Impact**						
Mean	1.78	1.92	−1.25	−0.24	0.50	0.01
SD	(1.41)	(0.88)	(0.19)	(0.43)	(0.50)	(0.98)

### Sociometric Status and Personal and Family Characteristics

The distribution by gender among the different statuses showed significant differences, χ^2^(4) = 21.084, *p* < 0.001 (Table 3). The proportion of boys in the rejected and neglected status was much higher than expected, and that of girls was higher in the average and popular status (adjusted standardized residuals > 2).

**TABLE 3:** Personal and socio-family characteristics of the sociometric status.

		Rejected	Controversial	Neglected	Average	Popular	Total
**Gender**	Male	68.5% (2.7)	65.6% (1.4)	70.9% (2.7)	51.0% (−2.3)	41.6% (−2.2)	53.5%
	Female	31.5% (−2.7)	34.4% (−1.4)	29.1% (−2.7)	49.0% (2.3)	58.4% (2.2)	46.5%
**Age**	Mean (SD)	12.63 (0.80)	12.50 (0.76)	12.36 (0.64)	12.46 (0.70)	12.30 (0.58)	12.45 (0.70)
**CSR**	Mean (SD)	0.35 (0.69)	0.21 (0.49)	0.07 (0.26)	0.19 (0.51)	0.07 (0.26)	0.18 (0.50)

*CSR, Cumulative Socio-Family Risk Index. Below the percentages, in parentheses, are the Adjusted Standardized Residuals.*

Regarding age, some differences were close to statistical significance, *F*(4, 826) = 2.34, *p* = 0.054. The popular adolescents were younger on average, closely followed by the neglected ones. The average and the controversial adolescents were in an intermediate position, with the rejected ones being the oldest.

The presence of certain family variables (addictions, economic needs, unstructured family, and a different ethnic group) was analyzed among the students of each of the sociometric statuses. Although their presence was always greater in the group of rejected boys, the group differences were only significant in unstructured families, χ^2^(4) = 16.38, *p* < 0.01. The percentage of rejected adolescents living in unstructured families was higher than in the other groups (adjusted standardized residuals > 2). The neglected and popular students lived in unstructured families to a lesser extent.

When comparing the mean scores of the Cumulative Socio-Family Risk Index, the ANOVA yielded significant differences between the scores of the different groups, *F*(4, 826) = 3.71, *p* < 0.01. The highest Socio-Family Risk Index was found among the students in the rejected group, followed by the controversial group. The lowest Socio-Family Risk Index was observed in the neglected and popular groups. It was observed that the rejected group scored significantly higher than the neglected (*d* = 0.27) and popular groups (*d* = 0.27) on the Cumulative Socio-Family Risk Index, with small effect sizes.

### Sociometric Status and Teacher’s Perception of School Adjustment

The school adjustment of each student was evaluated through their tutors. On a scale of 1 to 10, the total mean score of the Teacher’s Perception of School Adjustment Scale was 6.49 (SD = 1.69). There were clear differences in the school adjustment of adolescents of the different sociometric statuses, *F*(4, 826) = 16.72, *p* < 0.001. The best school adjustment was presented by students of the popular status (*M* = 7.65, SD = 1.24). Bonferroni’s *post hoc* tests indicated that this score was higher than that of the students in the rest of the groups (*p* < 0.05).

**FIGURE 1:**
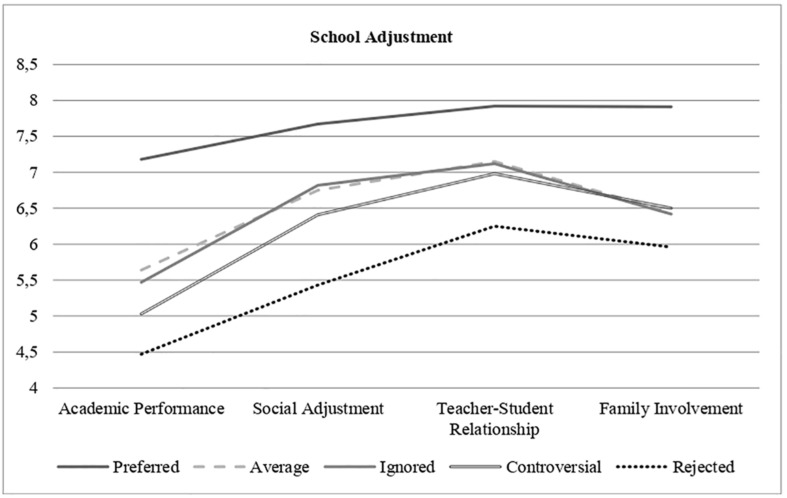
School adjustment and Sociometric status.

Regarding the subscales of the Teacher’s Perception of School Adjustment Scale, Academic Performance, *F*(4, 825) = 15.72, *p* < 0.001, Social Adjustment, *F*(4, 826) = 16.57, *p* < 0.001, Relations with Teachers, *F*(4, 825) = 10.19, *p* < 0.001, and Family Involvement, *F*(4, 803) = 8.07, *p* < 0.001 were significantly different among the five sociometric statuses. The highest scores of the popular adolescents and the lowest scores of the rejected adolescents stand out (Figure 1). Table 4 shows the magnitude of the comparisons.

**TABLE 4:** Cohen’s *d* value for *post hoc* contrasts between sociometric groups on EA-P dimensions.

R vs C	R vs N	R vs A	R vs P	C vs N	C vs A	C vs P	N vs A	N vs P	A vs P
EA-P	0.55	0.57	1.27			0.84		0.71	0.69
AP		0.52	1.20			0.68		0.75	0.68
FI			0.82			0.59		0.63	0.62
SA	0.78	0.74	1.25			0.71			0.51
TSR	0.52	0.54	1.01					0.48	0.46

*R, rejected; C, controversial; N, neglected; A, average; P, popular; EA-P, Scale of Teacher’s Perception of School Adjustment; AP, academic performance; FI, family involvement; SA, social adjustment; TSR, teacher–student relationship.*

### Predictive Capacity of Personal, Socio-Family, and School Adjustment Variables for the Social Preference Index

A linear regression analysis was performed to study the effect of personal and socio-family variables and the Teacher’s Perception of School Adjustment on social preference. To confirm the validity of the model, we analyzed the independence of the residuals. The Durbin-Watson statistic obtained a value of *D* = 2.073, confirming the absence of positive (values close to 0) and negative (values close to 4) autocorrelations. The absence of collinearity was also assumed and, thus, the stability of the estimates when obtaining high tolerance values and low Variance Inflation Factors (VIF).

In the first step, gender (coded as boy = 1, girl = 0), age, and CSR index were entered and, in the second step, three subscales of the Teacher’s Perception of School Adjustment were introduced (Academic Performance, Teacher–Student Relationship, and Family Involvement).

The regression model was significant and predicted 14.5% of the variance of social preference (Table 5). The slope of the preference among classmates decreased in boys, older students, and students with higher CSR as the values of academic performance, relationship with teachers, and family involvement decreased.

**TABLE 5:** Regression analysis to predict Social Preference Index based on personal, socio-family, and school adaptation variables.

Variables	*R*	*R*^2^	Adjusted *R*^2^	SE	β	Collinearity statistics	
						Tolerance VIF	
Gender	0.173	0.030	0.029	0.977	−0.119**	0.972	1.029
CSR	0.216	0.047	0.044	0.970	−0.043*	0.842	1.187
Age	0.236	0.056	0.049	0.966	–0.015	0.917	1.090
Academic achievement	0.356	0.127	0.122	0.929	0.268**	0.404	2.476
Student-teacher relationship	0.374	0.140	0.134	0.923	0.175**	0.572	1.747
Family involvement	0.381	0.145	0.139	0.920	0.111*	0.440	2.273

CSR, Cumulative Socio-Family Risk Index; VIF, Variance Inflation Factor. *p < 0.05. **p < 0.001.

### Predictive Model of the Social Preference Index

We used an SEM to provide an overview of interrelationships and influences of the variables (personal, socio-family risk, and school adjustment variables) that better explain adolescents’ social preferences. First, the normality of the data, skewness and kurtosis, and the multivariate kurtosis Mardia coefficient were analyzed. In our structural analysis, the Mardia coefficient was 23.31, and the normalized estimate was 29.50, exceeding by far the limit value of 5 established to be considered a multivariate normal distribution (Bentler, 2005). Therefore, the Robust Maximum Likelihood method was used.

The relationship between the variables showed the importance of the variables included in the model, whose standardized regression coefficients revealed their influence in the dependent latent variable. Given the sensitivity of the chi-square statistic to sample size, additional measures of model fit were used: the root mean square error of approximation (RMSEA), the Bentler comparative fit index (CFI), the Bentler-Bonett non-normed fit index (NNFI), Bollen’s fit index (IFI), and McDonald’s fit index (MFI). All estimates were statistically significant, and the model fit adequately, as all the values of the global fit indices of the model met the criterion of being greater than or equal to 0.95, and the value of RMSEA was less than or equal to 0.08 (Ferrando and Anguiano-Carrasco, 2010; Ruiz et al., 2010). The fit indices obtained were: χ^2^ = 9.0830, *df* = 8, *p* = 0.335, RMSEA = 0.013, NNFI = 0.997, CFI = 0.999, IFI = 0.999, and MFI = 0.999.

**FIGURE 2:**
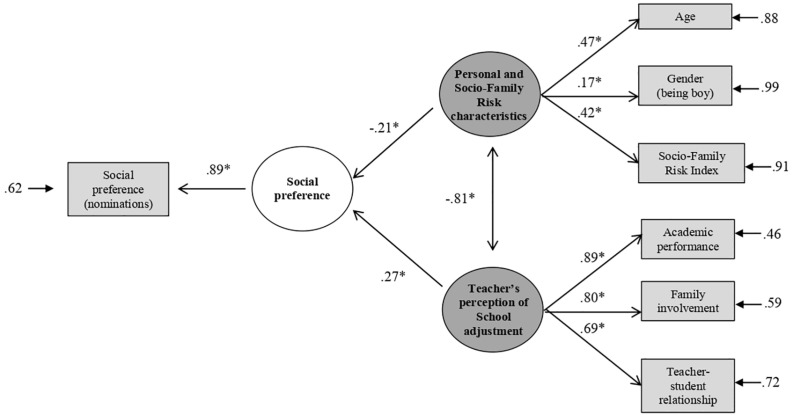
Model of structural equations social preference. *p < 0.05.

Figure 2 shows the variables included in the model and the standardized factorial coefficients. The indicators presented adequate factor loadings, ranging between 0.17 and 0.89. Personal and Socio-Family Risk characteristics, as well as School Adjustment (although to a lesser extent) had a direct relationship with social preference. These relationships accounted for 21% of the total variance of social preference. The model also revealed the existence of the interaction between the factors of personal and socio-family risk characteristics and academic adjustment.

## Discussion

The objective of this work was to identify personal, socio-family, and academic indicators related to the peer preference of Spanish adolescents in their classroom. Previous research has contributed to the knowledge of behavioral differences shown by children of different statuses. Less is known about the role of school adjustment and other personal and social characteristics of those involved. This work aimed to contribute in this regard. In this study, the sociometric technique was used to analyze social relationships in the classroom due to its easy application and empirical validity. The extensive sample of participants has allowed us to identify different statuses in each classroom, and discriminate some more prototypical personal, family, and school characteristics of each sociometric typology.

The results support the first hypothesis proposed in this work and coincide with those of other works (García-Bacete et al., 2010): Academic adjustment is positively related to a higher social preference among peers. The preferred adolescents receive a more positive academic evaluation by their teachers. They stand out for their better performance and school effort, presenting much higher scores than the other groups. They are also the youngest in the group, which denotes that they have seldom had to repeat a course. As Aparisi et al. (2015) pointed out, the children who are accepted the most by their peers are more interested in acquiring knowledge, as well as achieving good academic results, and advancing in their studies. Kingery et al. (2011), in a study with adolescents, also emphasized that those who get along the best with their classmates and are more accepted by their peers perform better in school. Good relationships with peers may be serving as a secure basis for exploration in the school setting (Meeus et al., 2002). At the opposite end, our results showed that rejected boys not only have difficulties at the social level but also present worse academic adjustment. This has been demonstrated in other studies where rejected adolescents are negatively valued by their teachers in variables such as acceptance, adaptation, effort, collaboration, participation, behavior, maturity, performance, intelligence, and success (García-Bacete et al., 1990). Also, these youngsters are somewhat older than the rest, probably because there is a higher proportion of children who have had to repeat some course. In the same line, other authors (Rodríguez et al., 2012) indicated that rejected adolescents report greater academic difficulties, more school failure, and less motivation towards studies than accepted adolescents. A review conducted by Wentzel et al. (2010) showed that adolescents who do not perceive their relationships with their classmates as being based on providing attention and support tend to present academic and behavioral problems.

Regression analysis and SEM confirm the strong relationships between children’s academic adjustment and social adjustment at the beginning of secondary education. These are probably two areas of personal adjustment where children are more or less successful in applying personal skills and knowledge. For both tasks, skills such as self-control, reflection, perspective-taking, or interpretation of rules are necessary. These skills may be poorly developed among children with worse social acceptance, who also fail to apply them to academic tasks. Deficits in information processing among rejected students have been reported by other researchers (Estévez et al., 2009). Systematic work on these competencies can therefore be useful to improve both the classroom climate and academic achievement.

Additionally, the results of this research highlight the two most opposing statuses, the children who received the most negative nominations (the rejected ones) and those who received the most positive nominations (the preferred ones), with the latter being the ones with the most differentiated profiles. Rejected children suffer the worst peer acceptance, and their tutors also describe their relationships with them as less positive. Our data show high correlations between teachers’ perception of children’s social adjustment and peer evaluation. Both tend to evaluate the same students negatively. The SEM corroborates this. As Cava and Musitu (2000) emphasized, the degree of coincidence between the perception of students and that of the teacher is usually high. Coinciding with other studies (Helsen et al., 2000; Cemalcilar, 2010), students who have non-conflictive relationships with the teacher are generally more accepted by their peers and are better adapted to school, unlike rejected children (García-Bacete and Monjas, 2009).

As the second hypothesis suggested, some personal and socio-family variables can act as risk factors for peer preference. The gender variable has shown significant differences in Social Preference, which is higher among girls. For example, there are more rejected boys than rejected girls. In both the Regression Analysis and the SEM, being a boy contributed to a lower peer preference. In this sense, Van de Schoot et al. (2010) also found more rejected (69.6 vs. 30.4%) and neglected boys (67 vs. 33%) compared to girls. Other previous studies also showed more favorable data for girls (Cava et al., 2010b; Tamm et al., 2014), which could be related to different patterns of socialization and different capacities for empathy in boys and girls (Mestre et al., 2009). Thus, for example, it has now been shown that adolescent girls need relationships with high emotional content earlier than boys (Naranjo Pou et al., 2020).

And finally, supporting Bronfenbrenner’s ecological model, some variables of the specific socio-family microsystem of adolescents appear to be related to peer preference. The social and family conditions of peer-rejected children seem to be more complicated, as indicated by the higher socio-family risk rate presented on average by this group. Drawing on the accumulated risk theory (Evans et al., 2013), the accumulation of stressors in these families, a lack of supports, and the practice of maladaptive educational patterns may be hindering the learning and development of socioemotional skills needed to establish and maintain adaptive peer relationships (Dishion, 1990; Evans and English, 2002). Studies with adolescents from at-risk families have precisely shown the implication of these family variables in the manifestation of externalizing and internalizing problems (Lorence et al., 2013), problems also closely related to peers’ greater or lesser preference. In this same line, the results of work carried out with other family variables (Tamm et al., 2014) have indicated that only the quality of the mother–child relationship was related to acceptance by adolescent peers. In this last study also, boys and girls who had more siblings, lived in a mono-maternal family, and had anxious attachment reported less acceptance by peers. Our results are in line with these other studies that show the link between risk variables from the family microsystem and the dynamics within peer groups.

The results of the subscale about parents’ participation in their children’s school life show that the families of adolescents with higher peer preference seem to be more involved in the school setting. There is less family involvement among the rejected children. In line with results obtained in other research, when tutors provide information about rejected adolescents, the teachers perceive their families as less participatory and less involved in the teaching and the educational community, with a lower degree of communication-agreement with the teachers and a lower cultural level. Some research has shown that this rejection profile may be determined by inappropriate relational styles learned within the family (Díaz-Aguado and Martínez, 2006; Estévez et al., 2007). As has been shown, and as other authors have been defending (Helsen et al., 2000), parent–child relationships have a great influence on adolescents’ behavior in other significant scenarios such as interaction with peers. According to the review of Martínez et al. (2012), parental support enhances children’s social competence by promoting their ability to develop positive social relationships, and this affects peer acceptance/rejection.

In conclusion, the data in this study support the hypotheses proposed at the beginning of the work. School adjustment is an important predictor of the success of social relationships among adolescent peers. Moreover, the presence of personal and socio-family risk variables makes it difficult for adolescents to be accepted by their peers, as formulated in the second hypothesis. We emphasize the need for school and family support to promote peer acceptance. Working on both aspects can help improve classroom coexistence. Given that the peer group does not always respond as expected to the rejected children’s change in behavior (Cava and Musitu, 2003), intervention techniques are recommended for the entire group, to intervene in attitudes, interpretations, and behaviors that enrich individual tools and the collective climate.

As a limitation to our work, we would like to point out that precisely the involvement of families in the academic monitoring of their children, as well as socio-family risk variables, were measured exclusively by the information provided by the tutors. Unlike teachers in early childhood education or primary education, secondary education tutors may not have sufficient information about these aspects. It would have been desirable to complete this information by other means. This lack of teachers’ knowledge of students’ family aspects may be indicating the importance of a mesosystem variable, such as the knowledge that teachers have of children’s family context of origin. As future lines of research, we aim to incorporate the direct evaluation of measures of relationship and involvement of the family context. It would also be interesting to know the expectations of teachers about their students in different fields (academic, personal, economic, and family well-being), which, in addition to conditioning the behavior of the students and affecting their academic evolution, could influence the parents’ perception of these students. Likewise, parents’ expectations about their children’s future are a key aspect to study for their adaptation and adjustment (Sánchez-Sandoval et al., 2019).

## Data Availability Statement

The raw data supporting the conclusions of this article will be made available by the authors, without undue reservation.

## Ethics Statement

The studies involving human participants were reviewed and approved by Comité Doctorado de la Universidad de Cádiz. Written informed consent to participate in this study was provided by the participants’ legal guardian/next of kin.

## Author Contributions

Both authors designed the study, collected and analyzed the data, and wrote the manuscript. Both authors contributed to the article and approved the submitted version.

## Conflict of Interest

The authors declare that the research was conducted in the absence of any commercial or financial relationships that could be construed as a potential conflict of interest.
